# Navigating Work and Aging in Older Adults with Sickle Cell Disease: Pursuits, Challenges, and Early Retirement

**DOI:** 10.1093/geroni/igaf122.2500

**Published:** 2025-12-31

**Authors:** Rania Mohamed, Tamara Poole, Francyess Denis Oliva, Teagan Callaway, John Strouse, Charity Oyedeji

**Affiliations:** Duke University School of Medicine, Durham, North Carolina, United States; Duke University School of Nursing, Durham, North Carolina, United States; Duke University School of Medicine, Durham, North Carolina, United States; Duke University School of Medicine, Durham, North Carolina, United States; Duke University School of Medicine, Durham, North Carolina, United States; Duke University School of Medicine, Durham, North Carolina, United States

## Abstract

Older adults with sickle cell disease (SCD) face age-related and SCD comorbidities leading to early functional decline, making it hard to work. This forces individuals to make tough decisions about their career ambitions. There is little research on work-related experiences of older adults with SCD. This study aims to depict work-related challenges faced by older adults with SCD.19 older adults with SCD were enrolled (age ≥ 50) and conducted semi-structured interviews about their experiences living with SCD and work-related obstacles. We used conventional content analysis to analyze transcripts. The mean age was 58 years (range 50-71) and 53% were male. Nearly half were externally employed (47%), 5% unemployed, 11% retired, 32% disabled, and 5% self-employed. There were three major themes. Theme 1 was “ Obstacles faced pursuing work “. Participants discussed barriers to advanced education, lack of resources to find jobs, rejection from jobs due to SCD, and narrowing job pursuits to those with limited physical demands. Theme 2 was “Challenges while working”. Participants discussed the complications, SCD pain, and physical limitations that interfered with work schedules and tasks. Participants described a lack of understanding from their employers and unreasonable expectations to immediately return to work following discharge from the hospital. Theme 3 was “Decisions to retire early”. Participants described needing to give up their business, retire early, or take periods of disability. This data from older adults with SCD revealed the need for greater awareness, support, and accommodation in the job market and workplace for individuals with SCD.

